# Structural evolution of CatSper1 in rodents is influenced by sperm competition, with effects on sperm swimming velocity

**DOI:** 10.1186/1471-2148-14-106

**Published:** 2014-05-16

**Authors:** Alberto Vicens, Maximiliano Tourmente, Eduardo RS Roldan

**Affiliations:** 1 Reproductive Ecology and Biology Group, Museo Nacional de Ciencias Naturales (CSIC), c/Jose Gutierrez Abascal 2, 28006 Madrid, Spain

**Keywords:** CatSper1, Sperm competition, Indels, Positive selection, Sperm velocity, Rodents

## Abstract

**Background:**

Competition between spermatozoa from rival males for success in fertilization (i.e., sperm competition) is an important selective force driving the evolution of male reproductive traits and promoting positive selection in genes related to reproductive function. Positive selection has been identified in reproductive proteins showing rapid divergence at nucleotide level. Other mutations, such as insertions and deletions (indels), also occur in protein-coding sequences. These structural changes, which exist in reproductive genes and result in length variation in coded proteins, could also be subjected to positive selection and be under the influence of sperm competition. *Catsper1* is one such reproductive gene coding for a germ-line specific voltage-gated calcium channel essential for sperm motility and fertilization. Positive selection appears to promote fixation of indels in the N-terminal region of CatSper1 in mammalian species. However, it is not known which selective forces underlie these changes and their implications for sperm function.

**Results:**

We tested if length variation in the N-terminal region of CatSper1 is influenced by sperm competition intensity in a group of closely related rodent species of the subfamily Murinae. Our results revealed a negative correlation between sequence length of CatSper1 and relative testes mass, a very good proxy of sperm competition levels. Since CatSper1 is important for sperm flagellar motility, we examined if length variation in the N-terminus of CatSper1 is linked to changes in sperm swimming velocity. We found a negative correlation between CatSper1 length and several sperm velocity parameters.

**Conclusions:**

Altogether, our results suggest that sperm competition selects for a shortening of the intracellular region of CatSper1 which, in turn, enhances sperm swimming velocity, an essential and adaptive trait for fertilization success.

## Background

Many genes involved in sexual reproduction evolve rapidly, often as a result of adaptive evolution or positive selection [1]. Several selective forces have been proposed to drive the adaptive evolution of reproductive genes, including sperm competition, female cryptic choice, or sexual conflict [2-4]. Sperm competition arises when multiple males copulate with a female in a polyandrous system. As a consequence, the ejaculate of each male competes with those of other males to be the first to fertilize the egg(s) [4,5]. Sperm competition generates postcopulatory sexual selection influencing reproductive traits to increase the success of an ejaculate at fertilizing under competitive conditions [4,6].

A critical determinant of the outcome of sperm competition is the relative number of spermatozoa provided by different males. Males respond to sperm competition by increasing sperm numbers, which is achieved by an increase in testes mass relative to body mass [4]. Relative testes mass associates to levels of sperm competition in many taxa [4,7-9] and, thus, is widely used as a reliable index of levels of sperm competition. Sperm swimming velocity is also a main determinant of fertilization success [10-13]. An increase in the size of sperm components is generally associated to increases in sperm swimming velocity [14-17].

Rapid divergence of reproductive proteins as a result of adaptive evolution may be linked to adaptive changes in reproductive traits under competitive conditions [1]. However, despite the potentially pervasive influence of sexual selection in driving adaptation at the molecular level, only a small set of studies has so far found an association between levels of sperm competition and rates of molecular evolution in reproductive genes [18-20]. In most studies searching for adaptively evolving proteins, the signature of positive selection has been tested estimating the ratio between nonsynonymous (amino acid-replacing) to synonymous (silent) nucleotide substitutions (dN/dS). Other forms of genetic variation different from nucleotide substitutions have received less attention in studies of adaptive evolution. One of these alternatives, i.e., structural variation, is widespread in animal genomes [21,22] and structural variants may shape the variation in phenotypic traits between individuals [23]. Therefore, positive selection might also act setting structural variants in gene coding sequences which may be advantageous for certain physiological traits. Insertions and deletions (indels) have been shown to be subjected to positive selection in *Drosophila* accessory gland proteins [24]*.* Indel substitutions are the most abundant structural variants in the mammalian genome [21] and previous studies have reported that some mammalian reproductive proteins show an elevated fixation of indels [25-27], suggesting that positive selection may play a role in the evolutionary change of protein length.

CatSper is a sperm-specific Ca^2+^ ion channel that is exclusively found in the plasma membrane of the principal piece of the mammalian sperm flagellum, and presumably forms a heterotetrameric, pH- and voltage-dependent Ca^2+^-permeable channel [28]. CatSper channel allows Ca^2+^ influx into the sperm flagellum, which is important for sperm motility at different stages in the life of spermatozoa during their transit along the female tract to the site of fertilization. Differences may exist between species with regards to the timing and roles of CatSper activation, or in the mechanisms underlying motility regulation [29-32]. Thus, some evidence suggests that CatSper may be related to motion through viscous media such as that observed in the cervix or, perhaps, the utero-tubal junction, but not in later events during transit in the oviduct [31], and that CatSper channels are key elements of major Ca^2+^ entry pathways during basal (the so-called activated) motility [30]. On the other hand, targeted disruption of CatSper channel subunits results in a lack of a more vigorous (so-called hyperactivated) motility in mouse spermatozoa after incubation in conditions that prepare them for fertilization (i.e., capacitation) [29]. Mutant male mice have spermatozoa with reduced sperm velocity parameters after incubations and they are infertile [29]. This has led to the conclusion that CatSper is essential for sperm hyperactivation, which takes place in the oviduct, and is necessary for ova penetration during fertilization [28]. It is possible that CatSper activation can elicit functionally different behaviors according to extracellular Ca^2+^ concentrations and to the sensitivity of the sperm Ca^2+^ stores [31]. Altogether, because CatSper is important for sperm motility, and is required for male fertility, this channel is a promising candidate for a key involvement in sperm competition.

A high number of indel substitutions have been favored by positive selection in the first exon of the *Catsper1* gene, which codes for the intracellular N-terminus of the CatSper channel [25,26]. Nevertheless, the selective forces underlying this high number of indels have not been identified. Although a clear function for this CatSper1 N-terminus has not yet emerged, it is possible that the length of this region might affect the regulation of the CatSper channel. In such case, the structural variation of the N-terminus of CatSper1 could affect sperm flagellar motility and, as a consequence, influence sperm swimming velocity, which is a major determinant of reproductive success in sperm competition.

In this study, we examined whether an elevated rate of indels in the N-terminus of the CatSper1 sequence has an adaptive value in terms of sperm competition in rodents. First, we tested whether the indel-related length variation of the N-terminal region of CatSper1 is associated to different levels of sperm competition. Second, given the vital role of the CatSper channel in sperm movement, and that sperm swimming speed is an important trait for fertilization, we assessed if changes in length of CatSper1 N-terminus are linked to phenotypic changes in sperm velocity parameters. Third, we analyzed whether sperm competition may promote episodes of positive selection at the nucleotide level. Fourth, since the amino terminus of CatSper1 may be involved in pH regulation of Catsper channel activity, due to its remarkably high content of histidine residues, we investigated whether the amount of histidines produced both by structural and molecular changes are associated with sperm competition and phenotypic adaptations. To this end, we sequenced the first exon of the *Catsper1* gene in several species belonging to the subfamily Murinae, assessed whether nucleotide and structural variations within this region may be driven by sperm competition, and examined possible associations between sequence length and sperm swimming velocity.

## Methods

### Species

Our study included a total of 16 rodent species belonging to the subfamily Murinae and comprising five genera: *Mus spretus, Mus spicilegus, Mus macedonicus, Mus famulus, Mus caroli, Mus cookii, Mus pahari, Mus m. musculus, Mus m. domesticus, Mus m.castaneus, Mus m. bactrianus, Mus minutoides, Mastomys natalensis, Apodemus sylvaticus, Lemniscomys barbarus* and *Rattus norvegicus*. This group of species covers a wide range of levels of sperm competition. Males of *Mus* species were purchased from the Institut des Sciences de l’Evolution, CNRS-Université Montpellier 2, France. *Apodemus sylvaticus* males were caught in the wild during the breeding session (Permit number 8688/02 from Consejería de Medio Ambiente, Comunidad de Madrid). Males of *Mastomys natalensis* and *Lemniscomys barbarus* come from wild-derived colonies which have been kept in captivity for only a few generations. Animal handling and housing followed the standards of the Spanish Animal Protection Regulation RD1201/2005, which conforms to European Union Regulation 2003/65. Animals were used complying with the Convention of Biological Diversity and the Convention on the Trade in Endangered Species of Wild Fauna and Flora. This study was approved by the Bioethics Committee of the Consejo Superior de Investigaciones Científicas (CSIC, Spain).

### *Catsper1* sequences

The first exon of *Catsper1* gene was amplified by polymerase chain reaction (PCR). PCR primers were designed based on the *Catsper1* sequences published in the NCBI GenBank (http://www.ncbi.nlm.nih.gov) for *Mus musculus* and *Rattus norvegicus* (accession numbers NM_139301 and XM_001070492, respectively) as well as the genomic data for multiple mouse strains available from the Sanger Institute database (http://www.sanger.ac.uk/). To design reverse primers, we also used *Catsper1* coding sequences reported previously [26] for *Mus* species (GenBank accession numbers DQ021482-DQ021500). These sequences were not employed to design forward primers because they started downstream of the start codon. PCR mixtures were prepared in a 50 μl volume containing PCR Gold buffer 1× (Roche, Barcelona, Spain), 2.5 mM MgCl_2_ (Roche), 0.8 mM dNTPs mix supplying 0.2 mM of each deoxinucleotide triphosphate (Applied-Biosystems, Barcelona, Spain), 0.3 mM of forward and reverse primers (Life Technologies, Madrid), 2 U of DNA polymerase (Biotools, Madrid), and 20–200 ng/μl of genomic DNA template. All PCRs were performed in a Veriti thermocycler (Applied-Biosystems). The conditions of the thermocycler program consisted of 35–45 cycles with an initial denaturation of 95°C for 30–40 s, an annealing stage at 58-62°C (depending on folding temperature of primers) for 60 s, and an elongation stage at 72°C for 80 s. PCR products were purified by using the E.Z.N.A.® Cycle Pure kit (Omega). Purified products were usually sequenced directly (Secugen S.L., Madrid, Spain). Products with problematic sequencing were cloned using pGEM®-T Vector System (Promega, Madrid, Spain) following the protocol provided by the manufacturer. The first exon of *Catsper1* was sequenced for at least 3 individuals per species in order to generate a consensus sequence. *Catsper1* sequences reported earlier [33] were not used to avoid polymorphisms due to different source populations.

### Alignments and trees

Processing and correction of sequences were performed using the sequence viewer and alignment editor BioEdit. Sequences from several individuals belonging to the same species were used to generate a consensus sequence per species. Consensus sequences were bound to the coding sequence using as reference the sequence of *Mus musculus* retrieved from NCBI GenBank (accession number NM_139301). Nucleotide sequences were aligned using the algorithm ClustalW implemented in BioEdit. To test the robustness of the alignment, we performed repetitive ClustalW varying penalty parameters for gap opening and gap extension. Those regions in which indels lead to inaccurate alignments were manually edited. Nucleotide coding sequences were translated to amino acid sequences and the correct frame was checked using the protein sequence of *Mus musculus,* retrieved from NCBI GenBank (accession number NP_647462), as well as those translated sequences from Podlaha et al. [33]. Amino acid sequences were aligned through ClustalW.

CatSper1 phylogenies were reconstructed using Neighbor-Joining (MEGA 5.03) and Maximum Likelihood (PhyML) methods. Statistical selection of best-fit model of nucleotide substitution was performed by JModelTest software [33]. For evolutionary analyses we used an input tree comprising our range of species on the basis of well resolved phylogenies for rodents (see Results section) [34-37].

### Tests for positive selection

We used the nonsynonymous/synonymous substitutions ratio (ω = dN/dS) as an indicator of selective pressure at the protein level, with ω = 1 indicating neutral evolution, ω < 1 purifying selection, and ω > 1 diversifying positive selection. To estimate rates of sequence evolution we used the application Codeml implemented in the PAML 4 package. In order to detect variable selective pressures in the first exon of *Catsper1* and infer residues under positive selection we applied models that account for heterogeneous ω ratios among amino acid sites [38]. We compared a null model that does not allow sites with ω >1 with a selection model that does through likelihood ratio tests. We used two kinds of likelihood ratio tests. The first compared a nearly neutral model M1a, which assumes values for ω between 0 and 1, with a model M2a which allows values of ω > 1. The second test is more refined and compares two models assuming a beta distribution for ω values. In this case, the null model M7 that limits ω between 0 and 1 is compared to the alternative model M8, that adds an extra class of sites with an ω ratio estimated to be greater than 1. We also used a third test comparing the M8 model with the null hypothesis M8a, which fixes ω to 1 instead of estimating an additional class of sites, reducing false positives [39]. These tests compare twice the log-likelihoods of the alternative and the null-model to critical values from a chi-square distribution with the degrees of freedom equal to the difference in the number of parameters between the two models. If the alternative models showed a significantly better fit in the likelihood-ratio-test, Bayes empirical Bayes (BEB) analysis [40] was used to infer positively selected sites with posterior probabilities higher than 0.95 under both model M2a and M8.

To adjust codon frequency and number of gamma categories, we ran repetitive analysis varying the values of these parameters and we used the setting with the best fit according to the likelihood values of models.

### Estimation of lineage-specific evolutionary rates

Site-analysis estimates variable ω ratios among sites and identifies residues under putative positive selection. Nonetheless, these models assume that the evolutionary parameters are invariant across the lineages of the phylogeny. We then calculated the evolutionary rates for each branch of the phylogeny using the Codeml free branch model [41]. Omegas were estimated for each lineage by adding dN and dS values from the root to the respective terminal branch and calculating the ratio of the sums. By calculating ω ratios from the root of the tree we considered the total accumulated selective pressures in Catsper1 during their evolution, which is more suitable for testing relationships against phenotypic data which do reflect the whole phenotypic evolution from the common ancestor [42]. In addition, estimating evolutionary data since the last common ancestor forces all branches to have the same length and therefore the analysis is not subject to temporal effects on dN/dS.

### Analysis of indel substitutions

Indels produced in the first exon of *Catsper1* were coded in the alignment by SeqState 1.41 software using a modified complex coding scheme. Events of indel substitutions were then inferred using the parsimony principle and considering the phylogenetic position of the species. This implies that, in cases where multiple equally parsimonious solutions for an indel were found, the first indel was assumed to happen in the split between the common ancestor of a clade with preponderance of an indel and the closest species carrying the indel variant. If the same indel variant was observed in any other species within the clade, such indel was considered homoplasious by evolutionary convergence. The species *Cricetulus griseus* was used as outgroup to infer indels occurring between *Rattus norvegicus* and remaining lineages. Indels were identified either as deletions or insertions and they were mapped onto the species tree (see Results section).

Length of the N-terminus of CatSper1 was calculated for each species as the total number of amino acids after checking that no indel is produced in the flanking regions and thus assuming that variations in sequence length are exclusively driven by internal indels.

### Analysis of histidines

Total number and proportion of histidines were calculated for each species to assess whether molecular and structural changes are promoting variations in the amount of this residue which is presumably important for the regulation of the CatSper channel.

### Sperm competition and sperm velocity parameters

Relative testes mass has been widely used as a reliable indicator of sperm competition levels in analyses of the evolution of ejaculate traits [9] and reproductive genes [18,20,43]. To obtain relative testes mass, males (N = 5 for each species) were sacrificed by cervical dislocation and weighed. After removal, the testes were weighed and measured. Values of relative testes mass for *Rattus norvegicus* were taken from the literature [44]. Mean relative testes mass values were calculated using the regression equation for rodents [44] (Additional file 1).

Sperm velocity parameters were measured using a computer-assisted sperm analyzer (Sperm Class Analyzer v.4.0, Microptic, Barcelona, Spain). A total of 5 μl of sperm suspensions was placed in a 20-μm deep slide chamber (Standard Count-2 Chamber Slide 20-micron, Leja, Nieuw-Vennep, Netherlands) pre-warmed to 37°C, and examined using phase contrast microscopy with a 4× objective. Data on sperm velocity parameters were obtained within 5 min of sample collection for all individuals.

Using a video camera (Basler A312fc, Vision Technologies), up to eight videos of 4 s each were recorded for each male’s sperm sample. Sperm concentration was previously adjusted to 4–6 × 10^6^ sperm/ml to satisfy the requirements of the analysis. Videos were analyzed and a minimum of 150 tracks were obtained for each male’s sample, with N = 5 males analyzed for each species.

Seven sperm velocity parameters were quantified: curvilinear velocity (VCL) (in μm/s), straight line velocity (VSL) (in μm/s), average path velocity (VAP) (in μm/s), linearity (LIN) (in%), straightness (STR) (in%), amplitude of lateral head displacement (ALH) (in μm/s) and beat cross frequency (BCF) (in Hz). To reduce potentially correlated components of velocity to a single factor summarizing the information, we performed a principal component analysis (PCA). The seven velocity descriptors were used as variables in the PCA, rendering two principal components that accounted for 70% (PC1) and 22% (PC2) of total variability. The three principal sperm velocity parameters (VCL, VSL, and VAP) showed a significant positive correlation with PC1 and no correlation with PC2, and thus PC1 was interpreted as the global measure of sperm velocity (hereafter referred as “overall sperm velocity”).

### Statistical analyses

Possible relationships between the evolution of the N-terminal region of CatSper 1 and phenotypic and ecological adaptations were evaluated through linear regression analyses. To test whether evolutionary dynamics of N-terminus of CatSper 1 are associated with sperm competition, linear regression analyses were performed using ω calculated from the root of the phylogeny and sequence length as dependent variables and relative testis mass as predictor variable. The same dependent variables were used in multiple regression analyses with body mass and testes mass as predictor variables. In this case, since predictor variables are related to each other, they were added to the multiple regression analysis in order, first body mass, and then testes mass, using a sequential (Type I) sum of squares. In order to search for relationships between CatSper 1 evolution and sperm velocity, different descriptors of sperm swimming velocity (see above) were used as dependent variables in simple regression analysis with ω and CatSper1 sequence length as predictor variables. The level of significance was adjusted to *P* < 0.05 for all tests. *Mus m.bactrianus, Mus famulus* and *Mus cookii* were not included in these analyses because of lack of data for these species.

Since species may share character values as a result of a common ancestry rather than independent evolution, regression analyses were performed using a phylogenetic generalized least-squares (PGLS) approach [45]. This powerful method allows for a control of phylogenetic effects on the associations between variables. PGLS analyses were conducted using the CAPER package for the statistical environment R v.2.10.1 (R Development Core Team, 2011). Phylogenetic effects were controlled based on the tree topology and branch lengths were calculated under the M0 model included in PAML.

## Results

### Catsper1 sequences

**Figure 1:**
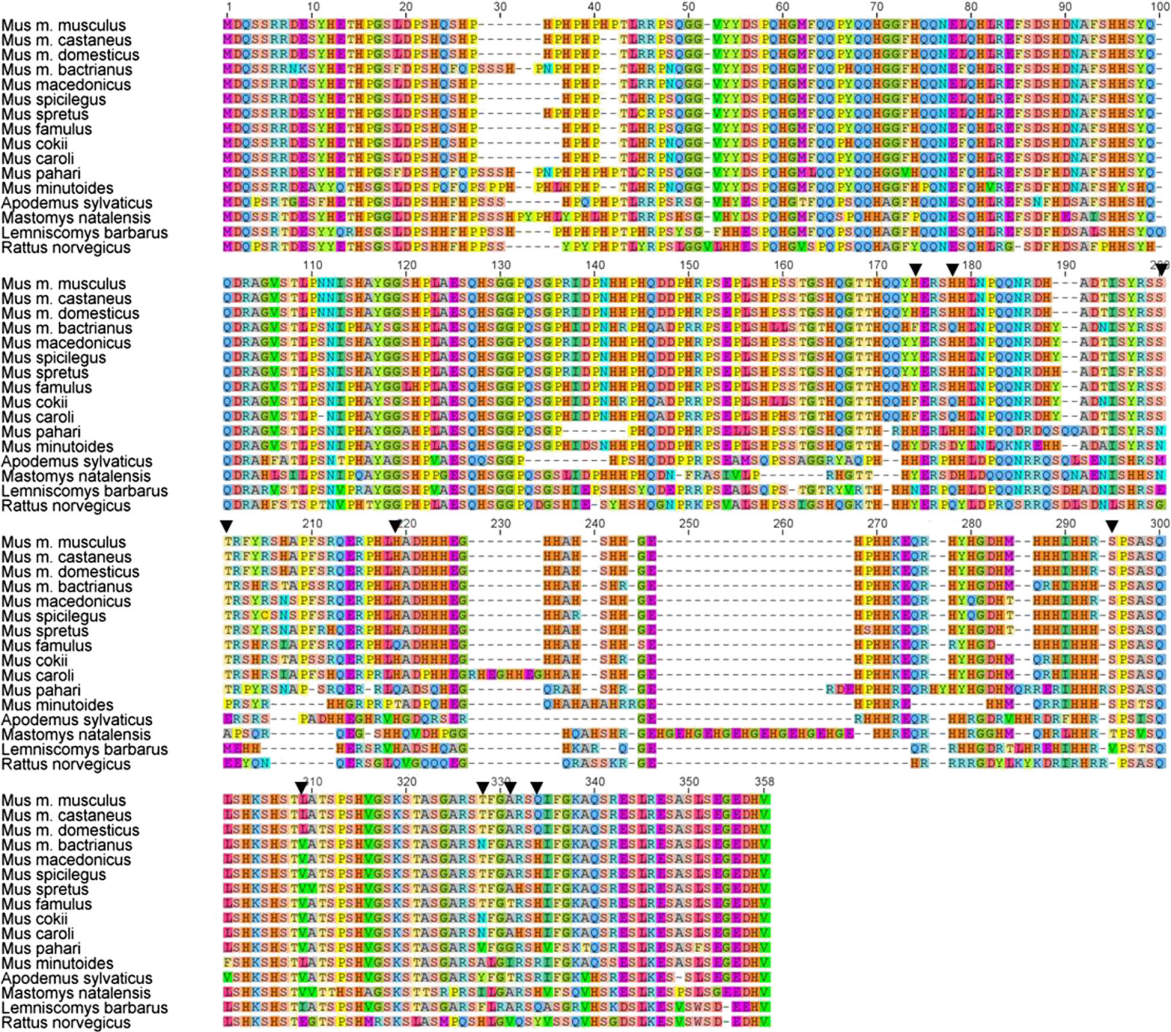
**Amino acid alignment of N-terminal region of CatSper1.** Translated sequences of 16 rodent species analyzed in this study. Dashes represent alignment gaps. Arrows on positions represent sites under putative positive selection with a Bayesian posterior probability >0.95 under M2a and M8 models.

Nucleotide sequences of the first exon of the *Catsper1* gene were obtained for 16 murid species and aligned according to their coding sequences. Multiple ClustalW alignment spanned 1080 nucleotides and revealed a high sequence divergence as well as an elevated number of gaps in *Catsper1* (see Additional file 2)*.* Sequences among species varied in length from 879 to 957 nucleotides. Amino acid alignment of the N-terminal region of CatSper1 is shown in Figure 1.

**Figure 2:**
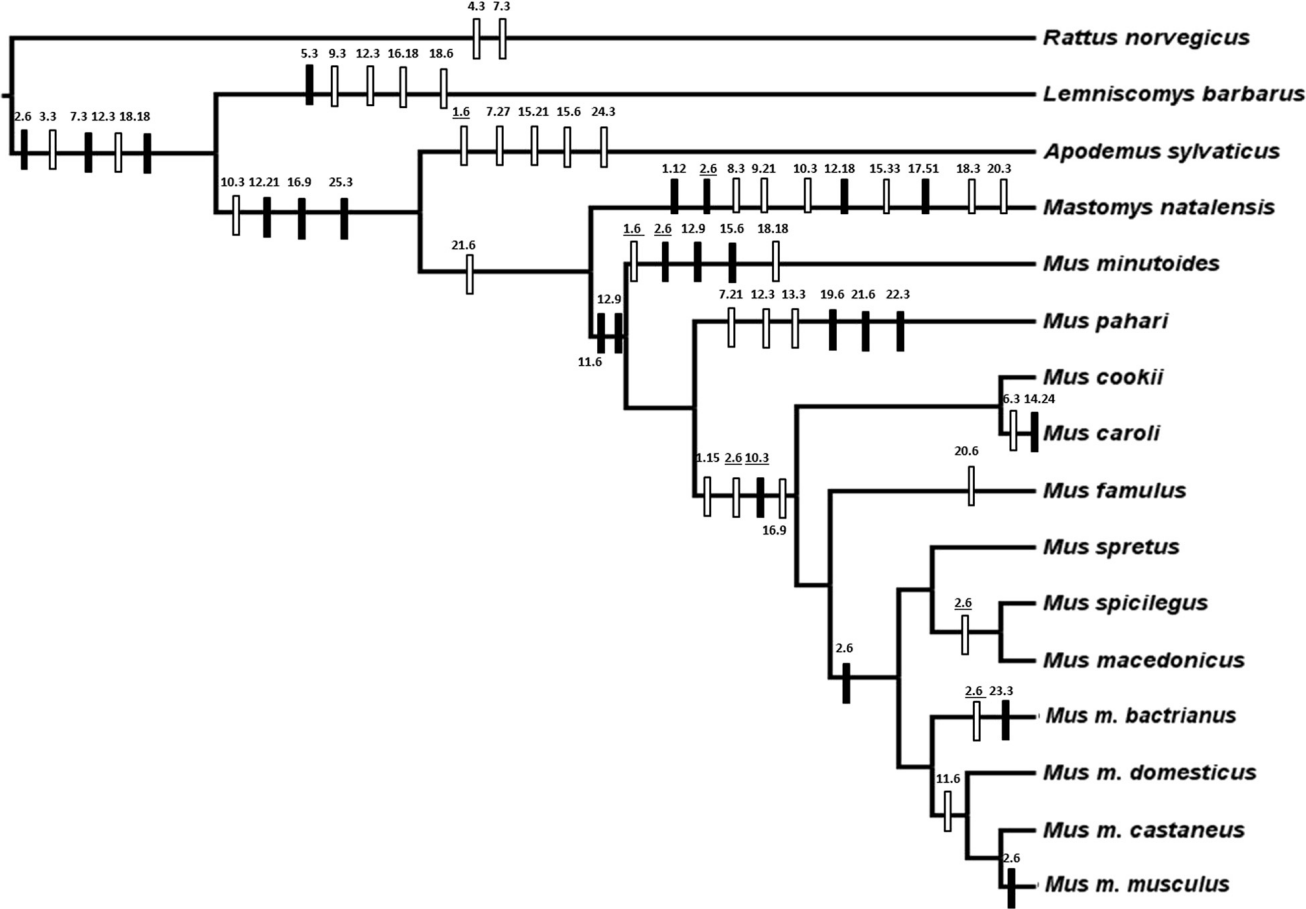
**Phylogeny of the 16 rodent species analyzed in this study.** Parsimony-informative indels are mapped throughout the tree. Filled rectangles indicate insertions and open rectangles indicate deletions. Indels are labeled according to region within the CatSper1 sequence where indels were identified (see Additional file 2: Figure S1) followed by number of inserted/deleted nucleotides. Underlined labels indicate homoplasious indels.

*Catsper1* phylogenies built by Neighbor-Joining and Maximum Likelihood approaches (Additional file 3) showed almost identical topology, but they showed some differences when compared to the species tree (Figure 2), which suggests that CatSper1 may be subjected to selective forces that would alter the evolutionary pattern expected from the phylogenetic relationships among the species. A total of 58 parsimony-inferred indel substitutions in the first exon of *Catsper1* were mapped onto the species tree (Figure 2). A total of 50 of these indels were unique and congruent with the tree topology and 8 were homoplasious (Figure 2). No indel polymorphism was found between individuals, by which we assume that all consensus sequences are representative of each species. A total of 35 indels fell in terminal branches and ranged from none for *Mus cokii, Mus spretus, Mus spicilegus, Mus macedonicus, Mus m. domesticus* and *Mus m. castaneus* to 9 for *Mastomys natalensis* (Figure 2)*.* A total of 34 deletions were detected throughout the phylogeny against 24 insertions.

All indels identified in the alignment spanned lengths of 3n nucleotides (see Figure 2 and Additional file 2), and thus the reading frame remained intact in all sequences. The number of observed indels in the first exon of *Catsper1* was significantly higher than both the genomic average and *Catsper1* locus neutral indel substitution rates (see [33] for methodology).

### Tests for positive selection

A high number of amino acid replacements was observed in CatSper1 N-terminus in addition to indels (Figure 1), suggesting that positive selection could also be promoting molecular variation in *Catsper1*. Robust evidence of positive selection was detected in the first exon of *Catsper1* when likelihood values of M2a and M8 selection models were compared with the corresponding values of M1a, M7 and M8a neutral models (Table 1). In both cases, likelihood ratio tests rejected the neutral models. A total of 10 and 9 significant positively selected sites were identified with a Bayesian posterior probability of *P*_B_ > 0.95 under M2a and M8 models respectively (Table 1, Figure 1). These analyses reveal that the N-terminal region of CatSper1 is subjected to strong positive selection also at the molecular level.

**Table 1:** Tests of positive selection in CatSper1

**N**	**Ls**	**Best fit model**	**Log-likelihood values**^**a**^	**Parameter estimates**	**PSS**^**b**^
16	235	M1a	-2907.9808	p_0_ = 0.269, p_1_ = 0.731, ω_0_ = 0.027, ω_1_ = 1	Not allowed
		M2a	-2879.567332 **	p_0_ = 0.186, p_1_ = 0.651 p_2_ = 0.163, ω_0_ = 0, ω_1_ = 1, ω_2_ = 4.67	124H*, 128H*, 146S*, 147 T**, 154H**, 165S*, 179 L*, 198 T**, 201A**, 204Q**
		M7	-2908.013308	p = 0.041, q = 0.012	Not allowed
		M8	-2879.676624 **	p_0_ = 0.833, p = 0.0166, q =0.0052, p_1_ = 0.166, ω = 4.535	124H*, 146S*, 147 T**, 154H**, 165S*, 179 L*, 198 T**, 201A**, 204Q**
		M8a	-2907.982933	p_0_ = 0.269, p = 2.758, q = 99.0, p_1_ = 0.731, ω = 1.0	Not allowed

^a^Differences between log-likelihood values of models with 99% statistical significance level for 2 degrees of freedom are shown as **.

^**b**^Positively selected sites (PSS) with a posterior probability >0.95 (*) and >0.99 (**) in Bayes Empirical Bayes.

N, Number of sequences aligned.

Ls, Sequence length (in codons) after alignment gaps are removed.

Parameter estimation and likelihood scores under site models.

We found that 4 out of 10 (40%) positively selected sites fell in positions containing histidines. Considering the possible role of histidine residues as a pH-sensor in CatSper1 activation, adaptive mutations on this domain may have important functional implications.

### CatSper1 evolution and sperm competition

Lineage-specific evolutionary rates (ω) estimated under PAML free branch model for the first exon of *Catsper1* were greater than 1 in all cases except for *Lemniscomys barbarus* (Additional file 1), thus revealing that intense positive selection is acting on CatSper among these rodent species. To seek evidence for an influence of sperm competition on the evolutionary rate of CatSper1, we correlated lineage-specific ω ratios with their respective relative testes mass values. Phylogenetic generalized least-squares (PGLS) analysis correcting for phylogenetic effects showed no significant associations of lineage-specific ω with relative testes mass or testes mass corrected for body mass (Table 2).

**Table 2:** PGLS analyses of evolutionary rate and sequence length of CatSper 1 in relation to relative testis mass (RTM)

**Dependent variable**	**Predictor**	**n**	**d.f.**	**Slope**	**R**^**2**^	**F**	**P**	**λ**^**a**^	**P**_**(λ=0)**_^**b**^	**P**_**(λ=1)**_^**b**^	**ES**^**c**^	**CL (-)**^**d**^	**CL (+)**^**d**^
Omega	RTM	16	14	-0.049	0.012	0.139	0.855	0.999	**< 0.001**	1	0.106	-0.438	0.649
Omega	Body mass	16	14	0.0022	0.539	13.939	0.0028	0.999	**<0.001**	1	0.052	-0.363	1.449
	Testes mass			-0.0924		0.14	0.7144				0.056	-0.439	0.647
Sequence length	RTM	15	13	-7.214	0.53	14.682	0.002**	0.402	0.257	0.078	0.925	**0.359**	**1.491**
Sequence length	Body mass	15	12	0.149	0.778	8.895	0.011*	0	1	**0.007**	0.779	**0.214**	**1.345**
	Testes mass			-21.159		33.055	0.0009**				1.28	**0.714**	**1.846**

*P* values < 0.05 (*) and < 0.01 (**) indicate statistical significance.

^a^λ value indicates phylogenetic effect.

^b^Significance of log-likelihood ratios for λ against models with λ = 0 and λ = 1 are shown. Statistically significant values are shown in bold.

^c^Effect size (ES) calculated from the *F*-values.

^d^Noncentral 95% confidence limits (CL). Confidence intervals excluding 0 indicate statistically significant relationships and are shown in bold.

**Figure 3:**
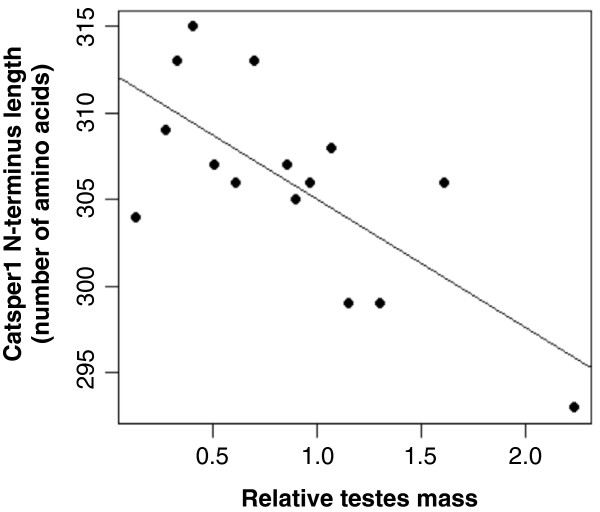
**Relationship between relative testes mass and the length of CatSper1 N-terminal region in rodents.** Results of statistical analyses are given in Table 2.

To test whether structural variation of CatSper1 N-terminal region is influenced by sperm competition, correlations between length of this region and relative testes mass were examined. Because the PGLS test is a least squares-based regression analysis, and it is highly sensitive to violations of assumptions and outliers, we first searched for the presence of outliers in our dataset. We found that sequence length of *Mastomys natalensis* was very different from that of the other species (Additional file 4). This was mainly due to its specific 51-bp long insertion produced as the result of a recent tandem duplication containing 3 codons (see Figure 1, Additional file 2). Therefore, we have not included this lineage in the analyses. A significant negative correlation was observed between the length of CatSper1 N-terminus and relative testes mass (Table 2). Species with high values of relative testes mass (i.e., with higher levels of sperm competition) presented a shorter N-terminus of CatSper1 whilst those with lower values of relative testes mass (low intensity of sperm competition) showed longer fragments (Figure 3).

### CatSper1 evolution and sperm swimming velocity

**Figure 4:**
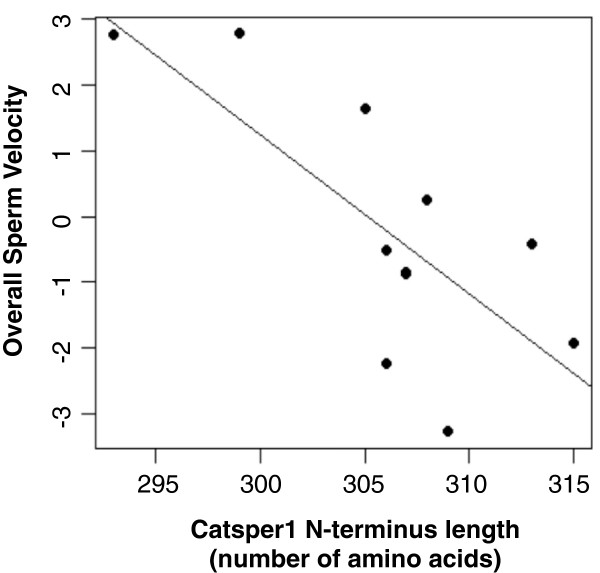
**Correlations between sequence length of the N-terminal region of CatSper1 with overall sperm velocity.** Results of statistical analyses for all velocity components are given in Table 3.

Given the important role of CatSper in sperm motility, we evaluated whether molecular and structural divergence of its N-intracellular domain has effects on sperm movement properties. No significant relationships were obtained when evolutionary rates of *Catsper1* were correlated with parameters of sperm velocity (data not shown). On the other hand, significant negative correlations were obtained when CatSper1 N-terminus length was analyzed with parameters measuring linear velocity (VSL, VAP, LIN, STR) and an overall sperm velocity component estimated from PCA (Table 3, Figure 4).

**Table 3:** PGLS of CatSper1 length in relation to sperm velocity parameters

**Dependent variable**	**Predictor**	**n**	**d.f.**	**Slope**	**R**^**2**^	**F**	**P**	**λ**^**a**^	**P**_**(λ=0)**_^**b**^	**P**_**(λ=1)**_^**b**^	**ES**^**c**^	**CL (-)**^**d**^	**CL (+)**^**d**^
VCL	Sequence length	11	9	-0.975	0.512	1.966	0.191	0.754	0.066	0.362	0.430	-0.223	1.083
VSL	Sequence length	11	9	-2.375	0.216	10.480	0.0089**	0.667	0.186	0.103	0.898	**0.245**	**1.551**
VAP	Sequence length	11	9	-2.003	0.164	8.340	0.016*	0.556	0.329	0.110	0.818	**0.165**	**1.472**
ALH	Sequence length	11	9	0.044	0.201	2.265	0.159	0.528	0.943	0.188	0.483	-0.21	1.176
LIN	Sequence length	11	9	-0.016	0.785	32.795	0.003***	0	1	**0.039**	1.402	**0.709**	**2.095**
STR	Sequence length	11	9	-0.009	0.626	15.06	0.001***	0.66	0.175	0.161	1.074	**0.381**	**1.767**
BCF	Sequence length	11	9	0.016	0.009	0.085	0.919	1	**0.001**	1	0.097	-0.595	0.789
Overall Sperm Velocity	Sequence length	11	9	-0.195	0.475	10.03	0.005**	0.652	0.128	0.154	0.920	**0.227**	**1.613**

*P* values < 0.05 (*), < 0.01 (**) and < 0.005 (***) indicate statistical significance.

^a^λ value indicates phylogenetic effect.

^b^Significance of log-likelihood ratios for λ against models with λ = 0 and λ = 1 are shown. Statistically significant values are shown in bold.

^c^Effect size (ES) calculated from the *F*-values.

^d^Noncentral 95% confidence limits (CL). Confidence intervals excluding 0 indicate statistically significant relationships and are shown in bold.

VCL, Curvilinear velocity; VSL, Straight-line velocity; VAP, Average path velocity; ALH, Amplitude of lateral head displacement; LIN, Linearity; STR, Straightness; BCF, Beat cross frequency.

### Changes in histidine residues

Because of its high histidine content, the N-intracellular region of CatSper1 is thought to be involved in the pH-mediated regulation of the CatSper channel. Therefore, we evaluated whether the variation in histidine content observed across rodent species is related to levels of sperm competition. We calculated the proportion of histidines in each sequence (Additional file 1) and performed PGLS analysis with relative testes mass as predictor; we found no significant correlation (*F* = 1.47, *P* = 0.25) (Additional file 5A). Likewise, no significant correlation was found between the proportion of histidines and parameters of sperm swimming velocity (data not shown).

These results suggest that sperm competition has no influence in histidine content of rodent CatSper1. No significant relationship was observed between sequence length and histidine proportion (*F* = 3.26, *P* = 0.0941). Thus, differences in histidine content are not attributable to residues gains and losses by insertions and deletions. Instead, two global histidine gains were detected throughout the phylogeny: the first occurring in the common ancestor of murid species after the split from rat, and a second between *Mus pahari* and the remaining *Mus* species (Additional file 5). These observations suggest that the proportion of histidines in CatSper1 is evolving following a phylogenetic pattern.

## Discussion

Previous studies in primates and rodents revealed that the rate of indel substitutions in the first exon of *Catsper1* is higher than that in neutral genomic regions, suggesting that positive selection is promoting length variation in CatSper1 [25,26]. Because CatSper1 is a possible candidate for direct involvement in sperm competition (see Background), we assessed whether the fixation of indel substitutions in the N-terminal region of CatSper1 is associated to this selective force. Our results revealed a significant negative correlation between the length of CatSper1 N-terminus and relative testes mass, a reliable proxy of sperm competition. This finding provides evidence that sperm competition has an influence on structural dynamics of CatSper and favors a shortening of the N-terminal domain of this protein. It has been reported that variation in molecular mass of SvsII, the major component of rodent copulatory plug, correlates positively with sperm competition levels across rodent species [27]. Given that most of the variation in the molecular mass of SvsII is presumably produced through insertions and deletions, this was so far the only evidence suggesting that indels may explain adaptive length divergence in reproductive proteins under scenarios of sperm competition. Our results now provide strong support for the idea that sexual selection is responsible for structural variation in some reproductive proteins.

The mechanisms generating insertions and deletions in the genome are not well understood. In any case, it has long been recognized that small deletions are more abundant than insertions of similar size in protein-coding sequences [46]. Our results agree with this evidence, because deletions outnumbered insertions in CatSper1. Moreover, we observed a variable number of indels across terminal branches, with some lineages showing insertion:deletion ratios that deviated from the expected ones. The most extreme cases were the species subjected to more intense sperm competition, namely *Apodemus sylvaticus* and *Lemniscomys barbarus* which showed almost an exclusive presence of deletions. It seems, therefore, that a higher fixation of deletions in the first exon of *Catsper1* is not only resulting from deletion bias as indel mechanism, but also that selective forces such as sperm competition are contributing to the shortening of this region.

CatSper1 is important for the regulation of intracellular Ca^2+^ required for both activated and hyperactivated sperm motility [29-31] that is needed at different stages during transit in the female tract. Experiments using null mice revealed that CatSper1 is essential for maintenance of sperm motility and hyperactivation [29,30], maintenance of intracellular ATP levels [47], progression beyond the oviductal sperm reservoir [48] and, ultimately, male fertility [29,49]. We thus tested whether length variation in the N-terminal region of CatSper1 is linked to sperm swimming velocity. Our results showed a significant negative correlation of CatSper1 N-terminus length with four parameters describing velocity (VSL, VAP, STR and LIN) and an overall sperm velocity component, revealing an association between the shortening of this region and increases in progressive motility. Our findings thus support a relationship between structural divergence of CatSper1 and sperm movement among rodent species. Previous studies have shown evidence that when sperm competition increases so does sperm swimming speed [14,17,50]. Swimming speed is a major determinant of fertilization success in sperm competition contexts [10,11]. Based on the correlation observed between relative testes mass, CatSper1 sequence length, and sperm velocity parameters, our results support the hypothesis that sperm competition favors shortening in the N-intracellular domain of CatSper1 leading to an increases of sperm swimming speed during transport of spermatozoa in the female reproductive tract and, as a consequence, increase the probabilities of fertilization success.

Although a high number of amino acid replacements, driven by positive selection, were observed in the N-terminal region of CatSper1, we did not find evidence of an association between ω ratios and levels of sperm competition or sperm velocity parameters. One possible interpretation of these results is that, whereas indel substitutions seem to be a primary target of post-copulatory sexual selection, amino acid divergence may be influenced by multiple selective forces promoting changes not related to reproductive success. On the other hand, it is possible that the different species follow different mutation routes to increase fertilization ability, leading to lineage heterogeneity of adaptive evolution, either within the *Catsper1* gene or across multiple genes controlling the same traits. Therefore, an effect of sexual selection on the molecular evolution of CatSper1 cannot be completely discarded despite the fact that our analyses did not reveal such an association.

The role of the N-intracellular region of CatSper1 has not yet been clarified. Catsper1 is a constitutively active unit of the CatSper channel, which is strongly potentiated by intracellular alkalinization [51]. An involvement of the CatSper1 N-terminal region in pH regulation of the CatSper channel has been suggested because of its high content in histidine residues [51]. Our results showed that variation in the proportion of histidines in the N-terminal region of rodent CatSper1 is not associated to sperm competition levels, sperm velocity or sequence length. Instead, we observed that different murine clades keep a constant amount of histidines, which suggests that successive histidine gains (or losses) have occurred through evolutionary time. Nonetheless, the acquisition (or loss) of histidines in CatSper1 could be adaptive as almost half of the amino acids under positive selection include these residues. On the other hand, previous reports have revealed that the sensitivity of K^+^ channels to intracellular pH is regulated by histidine residues located in particular intracellular domains [52,53]. It is therefore possible that pH sensitivity of CatSper1 is related to the location of histidine residues in the N-terminal domain rather than to their abundance.

Another suggested functional model for the intracellular N-terminal region of CatSper1 is one based on the ball-and-chain model of K^+^ channels [25]. This is plausible because the CatSper channel seems to resemble K^+^ channels rather than Ca^2+^ channels. According to the ball-and-chain model, the N-terminal domain of CatSper would act to physically block the ion channel pore region causing the inactivation of the channel. Variations in length of this intracellular domain could be relevant for the activation/inactivation rate of the channel due to spatial restrictions and, hence, may affect sperm motility [54]. In any case, there could be multiple mechanisms regulating Ca^2+^ influx through the CatSper channel and further molecular and biochemical approaches clarifying the role of the N-intracellular domain will be necessary to determine the functional consequences of indel and amino acid substitutions in this region as well as which changes may be advantageous in terms of sperm competition.

Our study provides the first evidence of how sperm competition is able to influence traits important for fertilization success by promoting structural changes in a sperm-specific protein. Sperm-specific proteins have evolved through large changes in protein length, with a larger number of indel events, in comparison with genes from other tissues in mammals [55]. Whenever indel substitutions do not imply drastic changes affecting protein function, structural divergence may be a source of variation able to promote advantageous changes more efficiently than nucleotide replacements. Therefore, it is possible that indel substitutions constitute primary targets of positive selection in some reproductive genes.

In any case, caution should be exercised when searching for links between genotypic and phenotypic traits. As mentioned above, in the context of sexual selection, species may exhibit several different mutational routes associated to increase in reproductive fitness. Moreover, many traits evolving under sexual selection are likely to be regulated by multiple genes. Analyses of groups of genes potentially associated with particular phenotypic traits could provide insights of the evolutionary processes of traits important for fertilization.

Our findings also contributed to identify a starting point for future work investigating the influence of evolutionary forces on structural divergence of protein coding sequences. It has been recently discovered that structural variants between close orthologous sequences are rare in the mouse and that a very low proportion of these lead to phenotypic changes [21]. Structural divergence is much more prevalent in duplicated genes, leading to the generation of functionally distinct paralogs [56]. A good example to address these issues may be CatSper itself and, thus, it could be worthwile expanding the present studies to other members of the CatSper gene family.

## Conclusions

Our study has revealed that length variation in the N-terminal region of CatSper1, resulting from an excess of indel substitutions favored by positive selection, is associated to relative testes mass, suggesting that sperm competition is a primary force influencing the structural evolution of this intracellular domain of the CatSper channel. In addition, our results have shown that variation in the length of CatSper1 N-terminus is associated with changes in sperm swimming velocity, an essential sperm function for successful fertilization. Altogether, the amino terminal region of CatSper1 seems to be an important target of sexual selection whose structural changes may result in faster sperm which are more likely to win the race to fertilization. To the best of our knowledge, this is the first observation of protein structural divergence linked to evolution of traits important for sperm competition.

## Availability of supporting data

CatSper1 sequences were deposited in GenBank (https://www.ncbi.nlm.nih.gov/genbank/) with accession numbers KJ652954-KJ652968. Catsper1 nucleotide matrices and resulting phylogenetic trees are available in the TreeBASE repository (http://www.treebase.org) with accession URL: http://purl.org/phylo/treebase/phylows/study/TB2:S15721 [57]. Supporting data are also included as additional files.

## Competing interests

The authors declare that they have no competing interests.

## Authors’ contributions

AV designed and performed the molecular experiments and analyzed data. MT performed the physiological experiments and obtained the phenotypic data. ERSR coordinated the study. AV, MT and ERSR wrote the paper. All authors read and approved the final manuscript.

## Supplementary Material

Additional file 1: Table S1 Data used for analyses. Click here for file

Additional file 2: Figure S1 Nucleotide alignment of first exon of *Catsper1*. Regions containing indel substitutions are indicated. *Cricetulus griseus* was used as outgroup. Click here for file

Additional file 3: Figure S2 *Catsper1* phylogenetic trees constructed by Neighbor-Joining and Maximum-likelihood methods. Click here for file

Additional file 4: Figure S3 Regression diagnostics between CatSper1 N-terminus length and relative testes mass. Click here for file

Additional file 5: Figure S4 Distribution of histidine abundance in CatSper 1 among rodent species**.** Click here for file
