# Supplementary material for: Structural evolution of CatSper1 in rodents is influenced by sperm competition, with effects on sperm swimming velocity
Source: BMC Evol Biol. 2014 May 16;14:106. doi: 10.1186/1471-2148-14-106 (PMC4041144; doi:10.1186/1471-2148-14-106)
Supplement: Additional file 1: Table S1 — Data used for analyses. [file 1471-2148-14-106-S1.pdf]

Supplementary table S1. Data used for analyses.

Species	Body Mass	Testes Mass	Relative Testes Mass	CatSper1 dN/dS (ω)	CatSper1 amino acids	Proportion of Histidines
Mus m. musculus	30.08	0.12	0.277	1.671	309	17.80
Mus m. castaneus	23.67	0.31	0.861	1.671	307	17.59
Mus m. bactrianus	16.36	0.14	0.506	1.467	313	16.61
Mus m. domesticus	19.50	0.10	0.328	1.671	307	17.59
Mus macedonicus	18.40	0.28	0.966	1.674	306	16.67
Mus spicilegus	15.14	0.41	1.612	1.674	306	16.67
Mus spretus	17.01	0.30	1.070	1.688	308	18.51
Mus famulus	27.40	0.05	0.134	1.628	304	17.76
Mus cokii	18.60	0.18	0.610	1.634	306	16.99
Mus caroli	7.95	0.11	0.700	1.633	313	18.21
Mus pahari	20.09	0.13	0.407	1.698	315	13.97
Mus minutoides	5.54	0.10	0.900	1.763	305	15.08
Mastomys natalensis	78.25	0.92	1.030	1.676	299	15.38
Apodemus sylvaticus	30.43	0.96	2.236	1.201	293	15.36
Lemniscomys barbarus	44.62	0.67	1.150	0.929	319	18.18
Rattus norvegicus	500.00	4.00	1.302	1.799	299	11.71
