# Supplementary material for: Structural evolution of CatSper1 in rodents is influenced by sperm competition, with effects on sperm swimming velocity
Source: BMC Evol Biol. 2014 May 16;14:106. doi: 10.1186/1471-2148-14-106 (PMC4041144; doi:10.1186/1471-2148-14-106)
Supplement: Additional file 2: Figure S1 — Nucleotide alignment of first exon of Catsper1. Regions containing indel substitutions are indicated. Cricetulus griseus was used as outgroup. [file 1471-2148-14-106-S2.pdf]

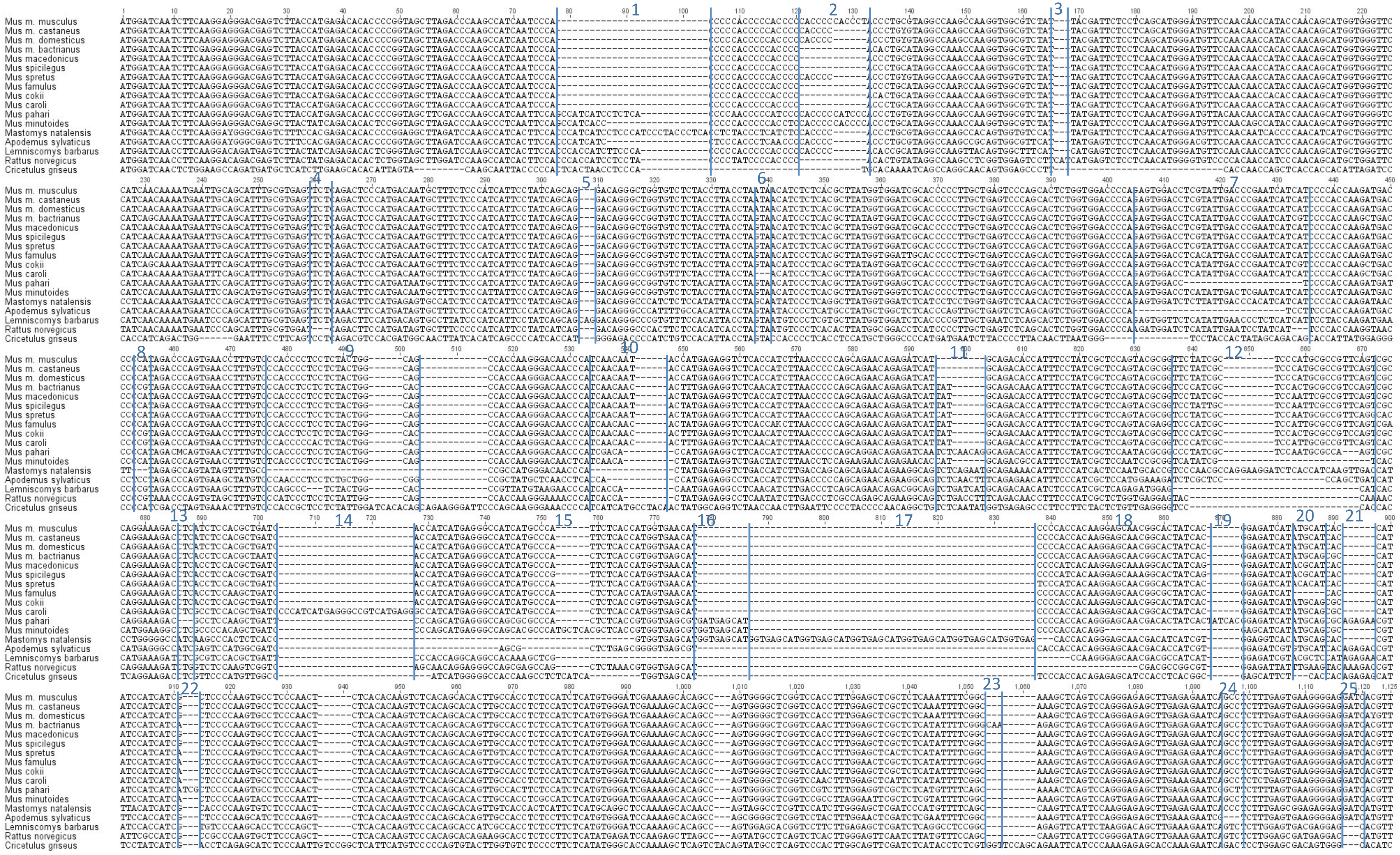

Figure S1 Nucleotide alignment of first exon of *Catsper1*. Regions containing indel substitutions are indicated. *Cricetulus griseus* was used as outgroup.
