# Supplementary material for: Structural evolution of CatSper1 in rodents is influenced by sperm competition, with effects on sperm swimming velocity
Source: BMC Evol Biol. 2014 May 16;14:106. doi: 10.1186/1471-2148-14-106 (PMC4041144; doi:10.1186/1471-2148-14-106)
Supplement: Additional file 3: Figure S2 — Catsper1 phylogenetic trees constructed by Neighbor-Joining and Maximum-likelihood methods. [file 1471-2148-14-106-S3.pdf]

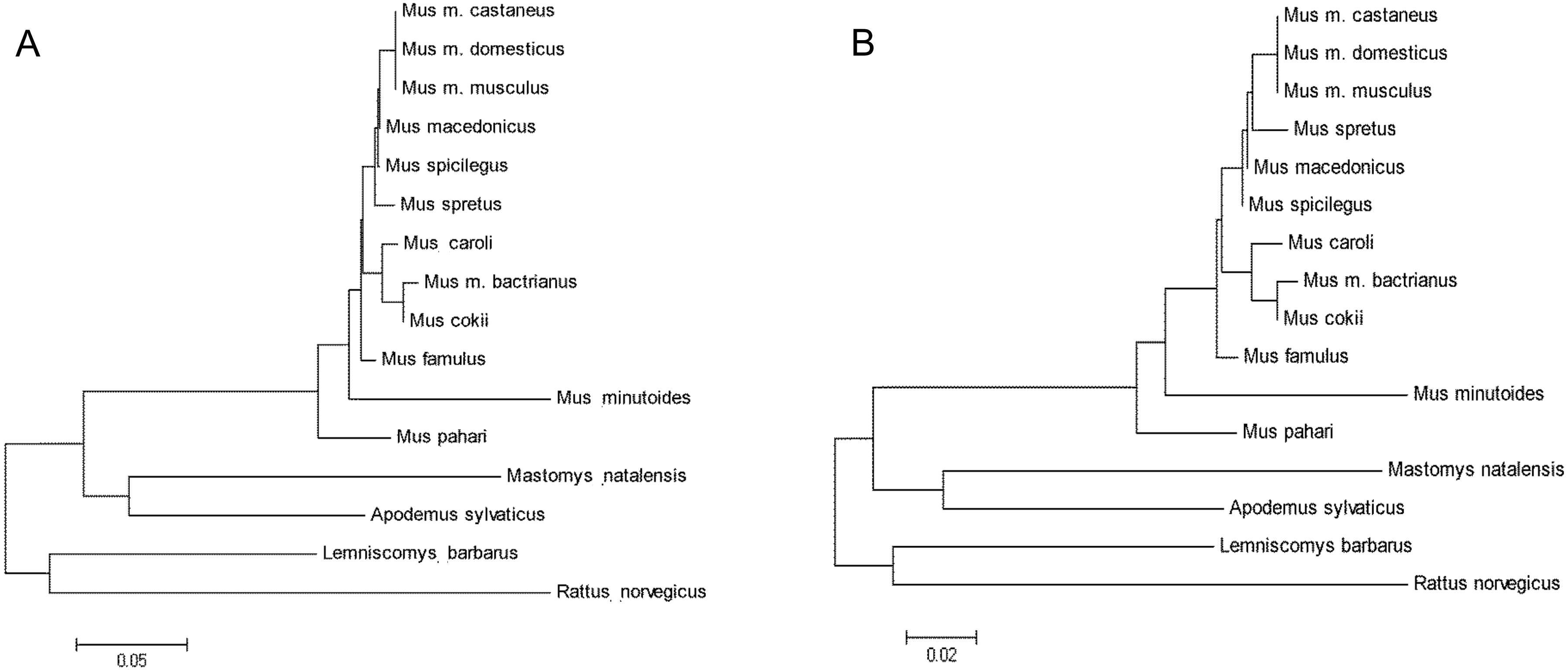

Figure S2 *CatSper1* phylogenetic trees constructed by **A**. Neighbor-Joining, and **B**. Maximum-likelihood methods. The tree is drawn to scale, with branch lengths in the same units as those of the evolutionary distances used to infer the phylogenetic tree.
