# Supplementary material for: Structural evolution of CatSper1 in rodents is influenced by sperm competition, with effects on sperm swimming velocity
Source: BMC Evol Biol. 2014 May 16;14:106. doi: 10.1186/1471-2148-14-106 (PMC4041144; doi:10.1186/1471-2148-14-106)
Supplement: Additional file 4: Figure S3 — Regression diagnostics between CatSper1 N-terminus length and relative testes mass. [file 1471-2148-14-106-S4.pdf]

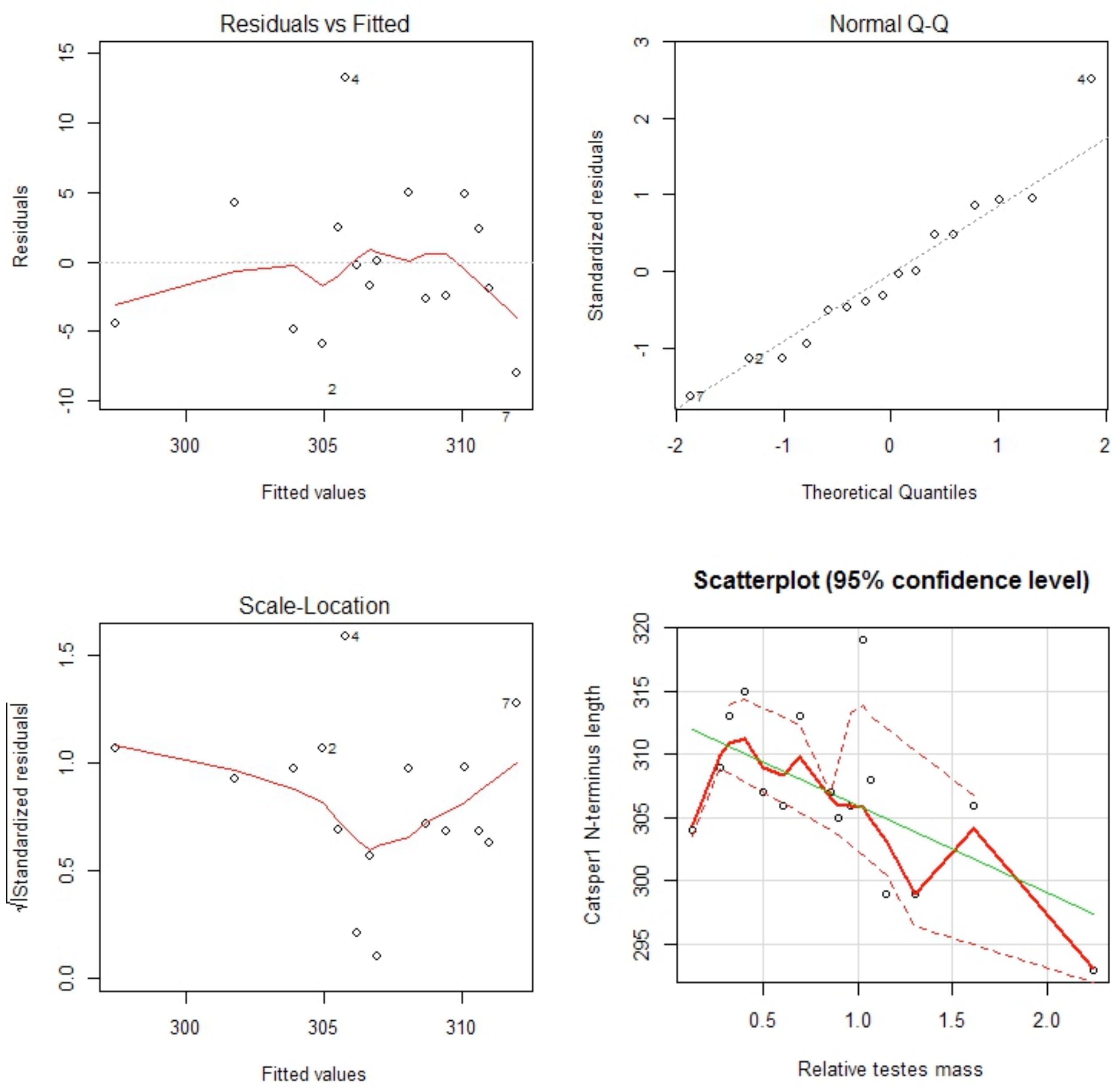

Figure S3 Regression diagnostics between CatSper1 N-terminus length and relative testes mass. Point number 4 is *Mastomys natalensis*, which was considered outlier by showing high deviation from the data distribution. Although not shown, we observed the same results for the regressions with sperm swimming speed variables.
