# Supplementary figures and images for: Structural evolution of CatSper1 in rodents is influenced by sperm competition, with effects on sperm swimming velocity
Source: BMC Evol Biol. 2014 May 16;14:106. doi: 10.1186/1471-2148-14-106 (PMC4041144; doi:10.1186/1471-2148-14-106)

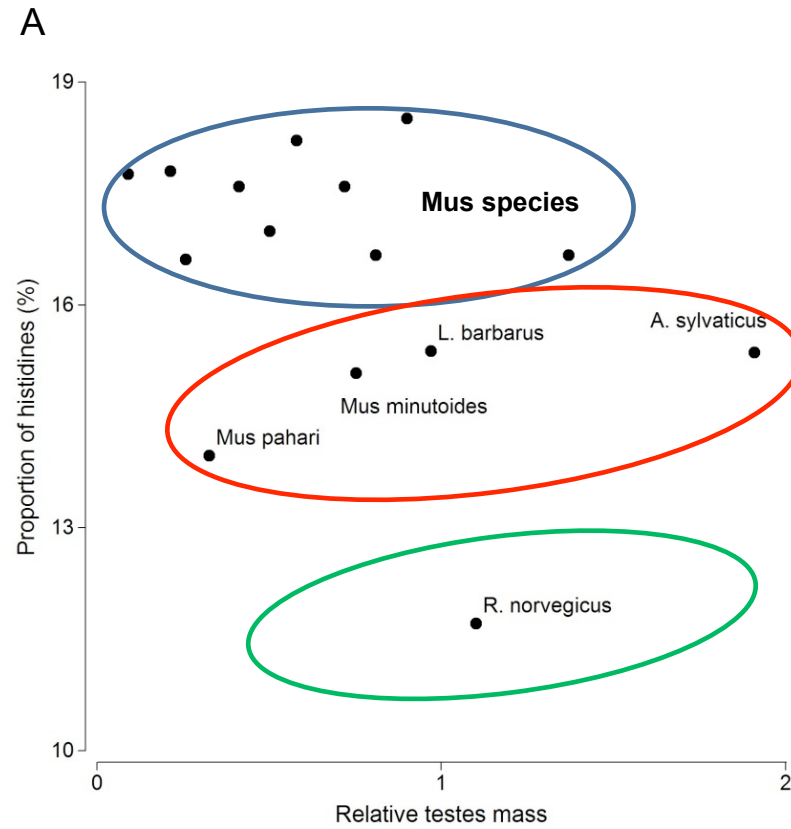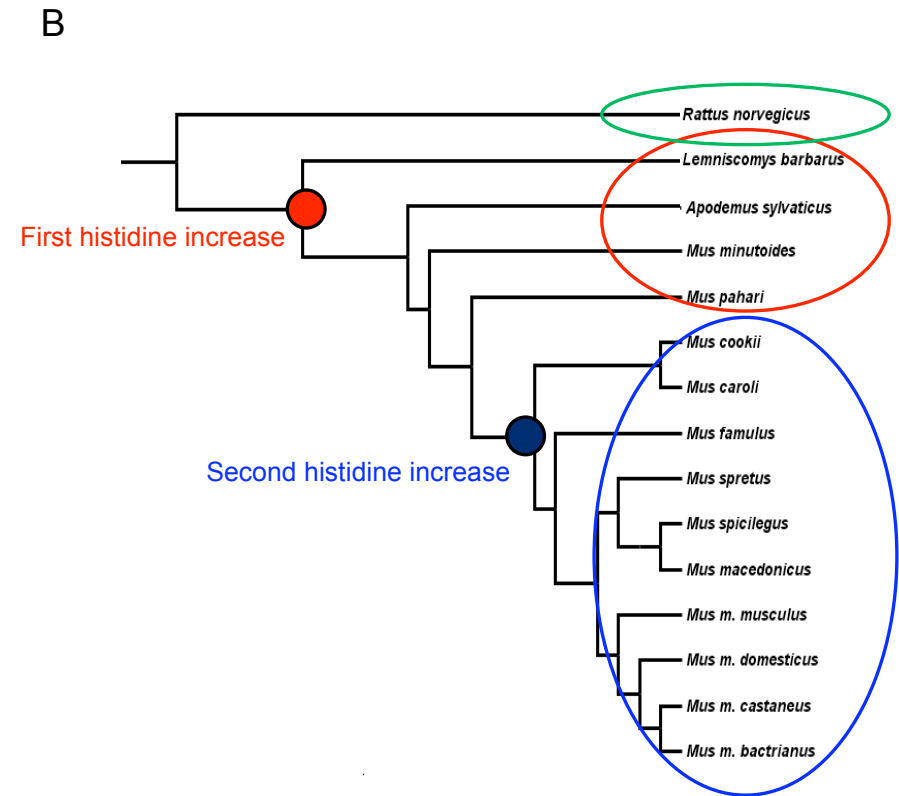

**Figure S4** Distribution of histidine abundance in CatSper1 among rodent species.

Supplement: Additional file 5: Figure S4 — Distribution of histidine abundance in CatSper 1 among rodent species. [file 1471-2148-14-106-S5.pdf]
